# A Network-Based Pharmacology Study of the Herb-Induced Liver Injury Potential of Traditional Hepatoprotective Chinese Herbal Medicines

**DOI:** 10.3390/molecules22040632

**Published:** 2017-04-14

**Authors:** Ming Hong, Sha Li, Hor Yue Tan, Fan Cheung, Ning Wang, Jihan Huang, Yibin Feng

**Affiliations:** 1 School of Chinese Medicine, Li Ka Shing Faculty of Medicine, The University of Hong Kong, Hong Kong, China; hong1986@connect.hku.hk (M.H.); lishasl0308@163.com (S.L.); hoeytan@connect.hku.hk (H.Y.T.); ofcheung@hku.hk (F.C.); ckwang@hku.hk (N.W.); 2 Shanghai University of Traditional Chinese Medicine, Shanghai, China; huangjihan@shutcm.edu.cn

**Keywords:** hepatoprotective Chinese herbal medicines, Xiao-Chai-Hu-Tang, *Radix Polygoni Multiflori*, Herb-Induced Liver Injury, network pharmacology

## Abstract

Herbal medicines are widely used for treating liver diseases and generally regarded as safe due to their extensive use in Traditional Chinese Medicine practice for thousands of years. However, in recent years, there have been increased concerns regarding the long-term risk of Herb-Induced Liver Injury (HILI) in patients with liver dysfunction. Herein, two representative Chinese herbal medicines: one—Xiao-Chai-Hu-Tang (XCHT)—a composite formula, and the other—*Radix Polygoni Multiflori (Heshouwu)*—a single herb, were analyzed by network pharmacology study. Based on the network pharmacology framework, we exploited the potential HILI effects of XCHT and *Heshouwu* by predicting the molecular mechanisms of HILI and identified the potential hepatotoxic ingredients in XCHT and *Heshouwu*. According to our network results, kaempferol and thymol in XCHT and rhein in *Heshouwu* exhibit the largest number of liver injury target connections, whereby CASP3, PPARG and MCL1 may be potential liver injury targets for these herbal medicines. This network pharmacology assay might serve as a useful tool to explore the underlying molecular mechanism of HILI. Based on the theoretical predictions, further experimental verification should be performed to validate the accuracy of the predicted interactions between herbal ingredients and protein targets in the future.

## 1. Introduction

Herb-Induced Liver injury (HILI) refers to herbal drug-driven liver injuries which are a cause of acute and chronic liver disease. Thousands of kinds of medicines have been implicated in causing various liver injuries and this is the most common reason for a drug to be withdrawn from the market [1].

Xiao-chai-hu-tang (XCHT), a Chinese herbal formula, is commonly used for treatment of chronic hepatitis and liver fibrosis in East Asian countries such as China, Japan and Korea. According to previous studies, XCHT exhibits various pharmacological properties including anti-inflammation, antioxidant and anti-hepatic fibrosis [2,3]. In addition, XCHT has shown strong hepatoprotective effects in both basic and clinical studies by regulating the immune response in the context of hepatitis C viral infection [4,5]. There are currently ongoing clinical trials of XCHT for hepatitis C and liver cirrhosis at the University of California, at San Diego and the Memorial Sloan-Kettering Cancer Center in the USA [5]. In spite of its multiple pharmacological effects, several systematic studies have examined and challenged its clinical safety. In 1995, four patients in Japan treated with XCHT by oral administration exhibited acute drug-induced liver injury [6]. In 2006, a case report in Taiwan also reminded us of the probable HILI potential of XCHT [7]. In 2000, the Japanese Ministry of Health Department completely forbade patients with hepatitis, liver cirrhosis and liver carcinoma to take XCHT [8]. The HILI of Radix *Polygoni Multiflori* (*Heshouwu* in Chinese) has also aroused widespread concern in recent years. *Heshouwu* has been used as a hepatoprotective and anti-aging tonic in Traditional Chinese Medicine (TCM) for thousands of years. In previous pharmacological studies, *Heshouwu* showed hepatoprotective effects against liver inflammation, oxidation stress, nonalcoholic fatty liver disease (NAFLD), liver fibrosis and cirrhosis as well as hepatic cancer [9,10]. However, increasing evidence also showed that *Heshouwu* might lead to significant adverse drug reactions in liver. Many case reports have revealed that long-term use of *Heshouwu* may induce toxic hepatitis or other severe liver injury, and even death [11,12,13]. Recent study has evaluated the “dose-time-toxicity” relationship of the HILI caused by *Heshouwu* in mice. The water-extracted components (from 5.5 to 30.75 g/kg) and the ethanol-extracted components (from 8.5 to 24.5 g/kg) caused obvious damage to the liver organization in a dose-dependent manner, resulting in increased serum alanine transaminase (ALT) and aspartate transaminase (AST) levels [14,15]. A compilation of liver injury cases have established causality for 28/57 different TCM herbal medicines, including *Heshouwu* and XCHT [14]. Although these studies have indicated that *Heshouwu* and XCHT may cause side effects on the liver, there has been insufficient scientific evidence to determine the HILI mechanisms of *Heshouwu* and XCHT.

In recent years, network pharmacology has made a significant contribution to investigating the molecular mechanisms of action of Chinese herbal medicines through chemical pharmacokinetic absorption, distribution, metabolism, excretion (ADME) property evaluation, target prediction and network/pathway analysis. Network pharmacology analysis plays an important role in the effective use of Chinese herbal medicines by predicating the potential toxic ingredients of candidate herbal drugs. Herein, based on the network pharmacology framework, we analyzed the potential HILI mechanisms of XCHT and *Heshouwu*. This network pharmacology technique could be of great help in predicting the safety of traditional herbal medicines and promoting the herbal medicine-based drug discovery.

## 2. Results and Discussion

### 2.1. Potential HILI Mechanisms of XCHT Predicted by Network Pharmacological Analysis

**Figure 1:**
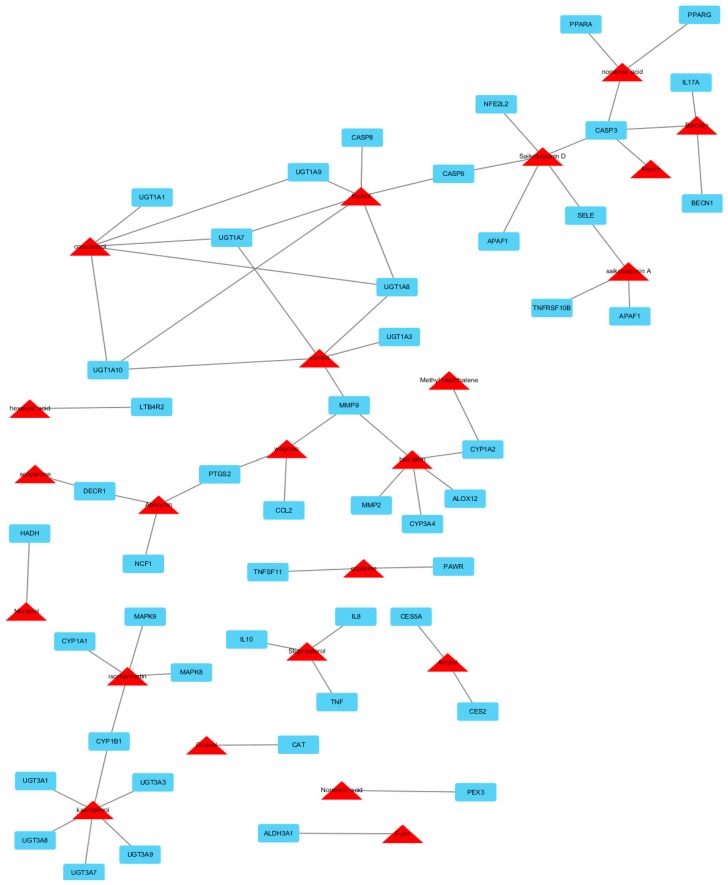
Compound-target hepatotoxicity network of Xiao chai hu tang. This network represents a global view of the potential compounds (red triangles) and targets (blue rectangles) in XCHT, and it comprised 74 nodes (23 potential compounds and 51 potential targets) and 131 edges (compound-target interactions).

We have identified a total of 761 chemicals in XCHT. In multi-compound medicinal herbal mixtures like XCHT, many compounds in that lack appropriate pharmaceutical properties are believed to fail to reach the cellular targets, and thus exhibit little efficacy and should be neglected. The 438 XCHT ingredients with satisfactory pharmacokinetic properties were predicted through an in silico-based pharmacological method (Supplemental Table S1). The compounds and protein interaction analysis results showed that a total of 676 intracellular targets were predicted to interact with the 438 ingredients of XCHT. Among them, 51 are regarded as potential HILI targets. To further predict and illuminate the relationships between these potential active compounds and hepatotoxic target genes, a drug-target network were constructed (Figure 1). This network represents a global view of the potential compounds (red triangles) and targets (blue rectangles) in XCHT, and it comprised 74 nodes (23 potential compounds and 51 potential targets) and 131 edges (compound-target interactions). The degree of nodes is a key topological parameter that characterizes the most influential nodes in a network, and we used it to further determine the importance of active components and HILI targets [16]. Those high-degree nodes in the network, which had more compound-target interactions, are likely to play a more important role in HILI [17,18]. Our network analysis results showed that various candidate compounds in XCHT were linked to multiple targets, which may exhibit potential liver injury effects. Among the 23 candidate compounds, kaempferol and thymol exhibit the largest number of liver injury targets connections (degree = 6), followed by saikosaponin D (degree = 5), coumestrol (degree = 5), vanillin (degree = 5) and baicalein (degree = 5). For the 51 potential liver injury targets, the network showed CASP3 had the largest number of compound-target interactions (saikosaponin D, baicalin, nonenoic acid and mairin), followed by UGT1A10, UGT1A7, UGT1A8 (vanillin, thymol and coumestrol), and MMP9 (vanillin, wogolin and baicalein). The remaining 46 targets showed interactions with only two or one compounds. The information of 51 potential liver injury targets in XCHT can be found in Table 1, where all the data were manually collected and integrated from the STITCH, TTD, PharmGKB and CTD databases.

Caspase 3 (CASP3), which had the largest number of compound interactions in our network pharmacology study, was indicated as the potential key target of XCHT-induced liver injury. Previous studies have indicated that CASP3 played an important role in liver diseases [19,20]. The hepatocyte apoptosis induced by TNF-α is correlated with the activation of CASP3 [21,22]. Both in vitro and in vivo studies confirmed that pancreatitis-associated ascitic fluid can induce hepatocyte apoptosis and liver injury by activating CASP3 dependent pro-apoptotic pathways [23]. In addition, lower expression of CASP3 can aggravate hepatic steatosis in NAFLD rat model by inducing hepatocyte apoptosis [24]. Thus, we predicted that CASP3 might be a potential target for inducing liver injury by XCHT. Saikosaponin D, baicalin, nonenoic acid and mairin from *Radix bupleuri* and *Radix scutellariae* were expected as the possible liver injury ingredients in XCHT by targeting CASP3.

Uridine 5′-diphospho-glucuronosyltransferase (UGT) is a crucial enzyme family in liver for detoxification and removal of exogenous compounds (e.g., dietary substances, drugs, toxins) and endogenous substances (e.g., bilirubin, steroid hormones, and bile acids) [25,26,27]. In our compound-target network, UGT also plays a pivotal role in XCHT-induced liver injury. Literature review showed that suppression of UGTs such as UGT1A10, UGT1A7, UGT1A8 by various endogenous or exogenous factors not only reduced the ability of certain drugs’ glucuronidation and thus triggered clinical adverse drug-drug interaction (clinical HILI), but also resulted in acute liver injury by metabolism dysfunction of endogenous substances [28,29,30].

Matrix metalloproteinase 9 (MMP9) also showed multiple target-compound interactions in our network study. Our in silico study showed MMP9 might play an important role in XCHT-induced liver injury by inducing interstitial fibrosis in liver. Previous studies showed that MMP9 could be prognostic markers for liver fibrosis [31]. MMP9 could modulate inflammation and extracellular matrix (ECM) remodeling in liver. In vitro studies showed that MMP9 activity was critical for TGFβ2-induced matrix contraction, and could promote hepatic fibrosis [32]. Down-regulation of MMP9 expression and the TGF-β1/Smad signaling pathways could relieve liver fibrosis in vivo [33]. Further mechanism studies demonstrated that during HILI process, Interleukin-1 produced by Kupffer cells or liver myofibroblast cells might play a pivotal role in remodeling of hepatic fibrosis through an either p38-dependent or p38-independent pathway to modulate the expression of MMP9 [34]. The above literature review studies can partly support our network pharmacology analysis results of XCHT.

### 2.2. Potential HILI Mechanisms of Heshouwu Predicted by Network Pharmacological Analysis

**Figure 2:**
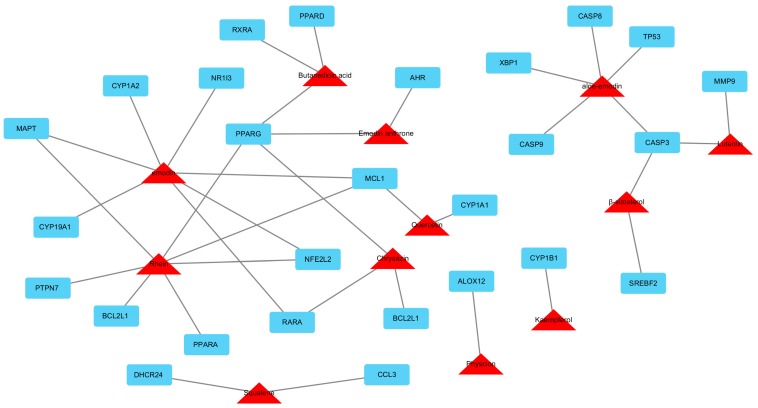
Compound-target network of *Heshouwu* that are associated with HILI. The red triangles are active compounds from *Heshouwu* and the blue rectangles represent potential hepatotoxic target genes, the grey lines represent the compound-target interaction. This network comprises 43 nodes (13 candidate compounds and 30 potential targets) and 62 edges (compound-target interactions).

We totally identified 73 major chemicals in *Heshouwu*. The sixteen ingredients with satisfactory pharmacokinetic properties of *Heshouwu* were predicted through an in silico-based pharmacologic method (Table 2). The compounds and proteins interaction analysis results showed that a total of 114 intracellular targets had potential interactions with the 16 ingredients of *Heshouwu*. Among them, 39 are regarded as potential HILI targets. To further predict and illuminate the relationship between these potential liver injury compounds and HILI target genes, a drug-target network was constructed (Figure 2). This network represents the interactions of the potential compounds (red triangles) and targets (blue rectangles) in *Heshouwu*, which comprised 43 nodes (13 candidate compounds and 30 potential targets) and 62 edges (compound-target interactions). Our network analysis results showed that various candidate compounds in *Heshouwu* were linked to multiple targets, which may exhibit multiple liver injury effects. Among the 13 candidate compounds, rhein exhibits the largest number of liver injury targets connections (degree = 7), followed by emodin (degree = 6) and aloe-emodin (degree = 5). For the 30 potential liver injury targets, the network showed that PPARG had the largest number of compound-target interactions (chrysazin, emodin anthrone, rhein and butanedioic acid), followed by CASP3 (aloe-emodin, β-sitosterol and luteolin), and MCL1 (emodin, rhein and quercetin). The remaining 27 targets showed interactions with only one or two compounds. The information of the 30 potential liver injury targets in *Heshouwu* is shown in Table 3, where all the data were manually collected and integrated from the STITCH, TTD, PharmGKB and CTD databases.

Interestingly, like XCHT, *Heshouwu* might also induce liver injury by targeting CASP3 according to our network analysis. In addition, peroxisome proliferator-activated receptor gamma (PPARG) and myeloid cell leukemia-1 (MCL1) are other two potential targets for *Heshouwu*-induced liver injury. PPARG, also known as the glitazone receptor, could regulate fatty acid storage and glucose metabolism. Inefficient fatty acid oxidation in mitochondria and increased oxidative damage are features of NAFLD. In rodent models and patients with NAFLD, hepatic expression of PPARG coactivator 1α (PPARGC1A) is inversely correlated with liver fat and disease severity. In mice, loss of estrogen signaling contributes to oxidative damage caused by low levels of PPARGC1A in liver, exacerbating steatohepatitis associated with high fructose and fat diets [35]. Furthermore, a clinical study also found that genetic variation in PPARG was associated with NAFLD, and the minor alleles haplotype was associated with inflammatory and fibrotic changes that denoted histologically advanced NAFLD [36]. MCL1, belonging to the Bcl-2 family, can enhance cell survival via inhibiting apoptosis [37]. Previous studies found that suppressing MCL1 could activate the mitochondrial apoptotic pathway in hepatocytes and induce tumorigenesis [38]. In addition, knockout of MCL1 can increase hepatic apoptosis in mice deregulating the expression of MCL1 may contribute to HILI [39]. The above statements partly support our network pharmacology analysis results of *Heshouwu*.

## 3. Materials and Methods

**Figure 3:**
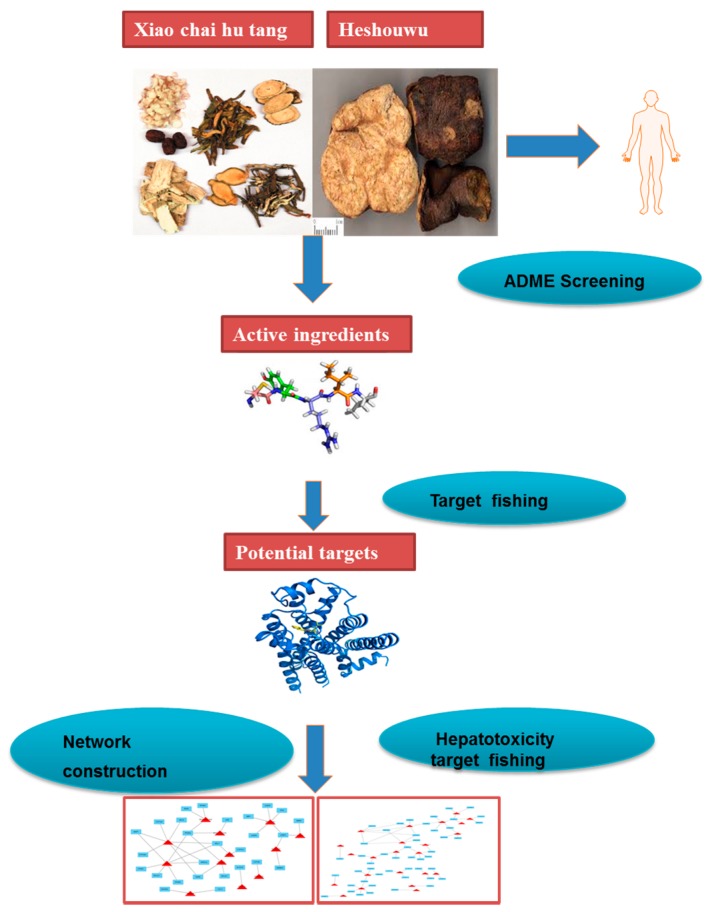
Network pharmacology approach workflow in this study.

The protocol of the integrated network pharmacology approach includes four main steps as follows (Figure 3).

### 3.1. Molecular Database Construction

All of the known ingredients of XCHT or *Heshouwu* were manually collected from related literature and two phytochemical databases: Traditional Chinese Medicine Systems Pharmacology Database (TCMSP, http://ibts.hkbu.edu.hk/LSP/tcmsp.php) and TCM Database@Taiwan (http://tcm.cmu.edu.tw/). The ingredients for these herbal medicines were retrieved from the above databases by using the following search terms: *Heshouwu*, Radix *Polygoni Multiflori*, xiao chai hu tang, Sho-saiko-to.

### 3.2. Pharmacokinetic ADME Evaluation

In this step, an in silico integrative model~ADME was used to select the ingredients with favorable pharmacokinetics properties. The ADME system used in this study including predict oral bioavailability (PreOB) and predict Caco-2 permeability (PreCaco-2). Oral bioavailability (OB) is one of the most vital pharmacokinetic properties of orally administered drugs as it plays an important role for the efficiency of the drug delivery to the systemic circulation [40,41] Here, a reliable in silico screening model (OBioavail 1.1) was employed in OB value calculation of the constituents in XCHT and *Heshouwu*. This model was constructed based on 805 structurally diverse drugs and drug-like molecules. Multiple linear regression, partial least square and support vector machine methods were applied during this model building, ending up with determination coefficient (*R*^2^) = 0.80 and standard error of estimate (*SEE*) = 0.31 for test sets [42,43]. In addition, for orally administered drugs, another pivotal problem is their movement across the intestinal epithelial barrier, which determines the rate and extent of human absorption and ultimately affects its bioavailability [44]. Thus, a preCaco-2 model was used to predict the drug absorption. The phytochemical information of the compounds with their Caco-2 permeability properties were explored using the TCMSP database, the detailed parameters’ information, screening criteria and calculation can be obtained from TCMSP website (http://ibts.hkbu.edu.hk/LSP/tcmsp.php). Finally, compounds with OB ≥ 33% and Caco2 ≥ 0.4 cm/s were regarded as active ingredients for further study. It is worth noting that the OB values of saikosaponin d and saikosaponin A are lower than 33%, but both of them are widely expected to induce liver injury in vitro and in vivo, thus, these two additional compounds were also regarded as candidate compounds [45].

### 3.3. Identification Targets for Potential HILI

In order to build the compound-target interaction profiles in XCHT and *Heshouwu*, we analyzed the chemical compounds and protein interactions integrated from the Search Tool for Interactions of Chemicals and Proteins (STITCH) 5.0 database (http://stitch.embl.de/) and Herbal Ingredients’ Targets (HIT) Database (http://lifecenter.sgst.cn/hit/), which are both databases of known and predicted interactions between chemicals and proteins. These databases were based on text mining and molecular docking technology to predict compound-protein interaction, which has been applied in the research of TCM for discovering potential active ingredients and interpreting molecular mechanisms of herbal medicines [46,47]. The STITCH 5.0 database can be used to study potential interactions between 300,000 phytochemicals and 2.6 million proteins curated from 1133 organisms. In this database, the approximate probability of a predicted association for a chemical–protein interaction is determined by the confidence score, with a higher score indicating a stronger interaction (low confidence score ~0.2; medium confidence score ~0.5; high confidence score ~0.75; highest confidence score ~0.95, provided by STITCH 5.0 database). The HIT database is a publicly available research resource that includes more than 116,000 interactions between 9300 chemicals and 13,300 genes. Both databases were searched independently by two researchers to minimize any bias. Then, to better define the HILI of XCHT or *Heshouwu*, Therapeutic Target Database (TTD, http://bidd.nus.edu.sg/group/ttd/), PharmGKB (http://www.pharmgkb.org) and Comparative Toxicogenomics Database (CTD, http://ctdbase.org/) [48,49,50] were employed to eliminate the noise and exclude irrelevant targets obtained from above steps. The targets for HILI were retrieved from above database by using the following search terms: Liver injury, hepatic damage, hepatic toxicity, hepatic drug metabolism, fatty liver, steatosis, lipid metabolism, liver fibrosis, oxidative damage, oxidative stress, liver inflammation, hepatocyte apoptosis, hepatocellular necrosis and hepatogenic jaundice. Only the targets of *Homo sapiens* were kept for further analysis.

### 3.4. Network Construction and Analzysis

To further explore the relationships between the compounds and targets associated with hepatotoxic effects, the Compound–Target network plotting was generated by Cytoscape 3.4.0 (http://www.cytoscape.org/) [51]. In the graphical network plot, nodes represent the compounds or proteins, and edges encode the compound–target interactions. In order to specify the importance of a node and how this node influences the communication between two nodes, all the properties of the network were analyzed by Network Analysis plugin.

## 4. Conclusions

Chinese herbal medicines are multi-component synergistic systems, which might play both therapeutic and toxic roles in humans. The HILI and quality control of herbal formulas or single herbs for public consumption are one of increasing concerns these days due to the lack of scientific evidence on their safety. In this paper, two drug-target networks of HILI of XCHT and *Heshouwu* were constructed through network pharmacology assays. The network predicted the potential underlying HILI mechanism of XCHT and *Heshouwu* and elucidated the interrelationship between hepatic toxicity and Chinese herbal medicine interventions. Therefore, network pharmacology is a useful tool to reveal the potential mechanism of HILI of Chinese herbal medicines and potential toxic ingredients. These data in our study, if combined with further in vitro and in vivo studies, should facilitate construction of a potential herb-induced liver injury network and improve the specificity of toxicity prediction for future drug candidates. Notwithstanding the advances in recent network pharmacology research, there are some crucial technical issues to be addressed and improved for data collection of herb-induced liver injury. First of all, the inventory of herbal products remains incomplete and new chemical structures are continuously being discovered. Secondly, previous researchers have explored and identified only a small part of the hepatotoxic target genes. Last but not least, stringently assessing the relationships between compounds and corresponding targets and obtaining accurate action modes such as activated drug-target interactions or inhibited drug-target interactions are still a challenge for present network pharmacology study. Despite these technical issues, for the multiple components-multiple targets interaction model of herbal medicines, conventional experimental research faces a situation of long-term investment to investigate the complex interaction mechanisms. Thus, our network pharmacology study which integrates the systems biology and in-silico technologies may offer a direction for the mechanism study of HILI. The high degree nodes, which may play hub roles in the compound-target network, will provide potential key object for next experimental study. Our results not only provide new insights for a deeper understanding of the molecular basis of potential HILI but also demonstrate a promising method for assessing the drug’s safety from herbal medicine. As further steps, herbal compound libraries should be established and further enriched to better correlate compound functions with structures. Experimental verification is also urgently needed to validate the accurate interactions between the predicted active ingredients and target proteins.

## Figures and Tables

**Table 1:** The information of 51 potential HILI targets in Xiao chai hu tang.

Target Gene	Target Protein	Biolocigcal Activity of the Targeted Proteins
DECR1	2,4-dienoyl CoA reductase 1	fatty acid-oxidation
UGT1A8	UDP glucuronosyltransferase 1 family, polypeptide A8	liver enzyme essential to the disposal of bilirubin
UGT1A7	UDP glucuronosyltransferase 1 family, polypeptide A7	liver enzyme essential to the disposal of bilirubin
CASP8	caspase 8	hepatocyte apoptosis
UGT1A9	UDP glucuronosyltransferase 1 family, polypeptide A9	liver enzyme essential to the disposal of bilirubin
UGT1A10	UDP glucuronosyltransferase 1 family, polypeptide A10	liver enzyme essential to the disposal of bilirubin
CASP9	caspase 9	hepatocyte apoptosis
LTB4R2	leukotriene B4 receptor 2	inflammation
NCF1	neutrophil cytosolic factor 1	oxidation stress
PTGS2	prostaglandin-endoperoxide synthase 2	inflammation
CAT	catalase	oxidation stress
CES5A	carboxylesterase 5A	metabolism of xenobiotics in liver
CES2	carboxylesterase 2	metabolism of xenobiotics in liver
CASP3	caspase 3	hepatocyte apoptosis
PPARG	peroxisome proliferator-activated receptorγ	oxidation stress
PPARA	peroxisome proliferator-activated receptorα	oxidation stress
IL10	interleukin 10	inflammation
TNF	tumor necrosis factor	hepatocyte apoptosis
IL8	interleukin 8	viral infection
HADH	hydroxyacyl-CoA dehydrogenase;	fatty acid-oxidation
CYP1A2	cytochrome P450, family 1, subfamily A, polypeptide 2	oxidation stress
PEX3	peroxisomal biogenesis factor 3	fatty acid-oxidation
CYP1B1	cytochrome P450, family 1, subfamily B, polypeptide 1	oxidation stress
CYP1A1	cytochrome P450, family 1, subfamily A, polypeptide 1	oxidation stress
MAPK8	mitogen-activated protein kinase 8	hepatocyte apoptosis
MAPK9	mitogen-activated protein kinase 9	hepatocyte apoptosis
UGT3A1	UDP glycosyltransferase 3 family, polypeptide A1	liver enzyme essential to the disposal of bilirubin
UGT3A3	UDP glycosyltransferase 3 family, polypeptide A3	liver enzyme essential to the disposal of bilirubin
UGT3A8	UDP glycosyltransferase 3 family, polypeptide A8	liver enzyme essential to the disposal of bilirubin
UGT3A7	UDP glycosyltransferase 3 family, polypeptide A7	liver enzyme essential to the disposal of bilirubin
UGT3A9	UDP glycosyltransferase 3 family, polypeptide A9	liver enzyme essential to the disposal of bilirubin
CASP3	caspase 3	hepatocyte apoptosis
NFE2L2	nuclear factor erythroid 2-related factor 2	oxidation stress
SELE	E-selectin precursor	hepatocyte apoptosis
APAF1	Apoptotic peptidase activating factor 1	hepatocyte apoptosis
TNFRSF10B	tumor necrosis factor receptor superfamily member 10B	hepatocyte apoptosis
ALDH3A1	aldehyde dehydrogenase 3 family, member A1;	detoxification of alcohol-derived acetaldehyde in liver
ALOX12	arachidonate 12-lipoxygenase	inflammation
MMP2	matrix metallopeptidase 2	liver fibrosis
MMP9	matrix metallopeptidase 9	liver fibrosis
CYP3A4	cytochrome P450, family 3, subfamily A, polypeptide 4	oxidation stress
BECN1	beclin 1	viral infection
IL17A	interleukin 17A	inflammation
CCL2	chemokine (C-C motif) ligand 2	inflammation
TNFSF11	tumor necrosis factor (ligand) superfamily, member 11	hepatocyte apoptosis
PAWR	PRKC, apoptosis, WT1, regulator	hepatocyte apoptosis
MMP9	matrix metallopeptidase 9	liver fibrosis
UGT1A3	UDP glucuronosyltransferase 1 family, polypeptide A3	liver enzyme essential to the disposal of bilirubin
UGT1A8	UDP glucuronosyltransferase 1 family, polypeptide A8	liver enzyme essential to the disposal of bilirubin
UGT1A9	UDP glucuronosyltransferase 1 family, polypeptide A9	liver enzyme essential to the disposal of bilirubin
UGT1A1	UDP glucuronosyltransferase 1 family, polypeptide A1	liver enzyme essential to the disposal of bilirubin

**Table 2:** Compounds with satisfactory pharmacokinetic properties of *Heshouwu*.

Molecule Name	Molecular Weight	Oral Bioavailability (%)	Predict Caco-2 Permeability
Physcion	284.28	32.29	0.52
Copaene	204.39	39.47	1.81
Luteolin	286.25	36.16	0.59
Quercetin	302.25	46.43	0.5
hexanoic acid	116.18	73.08	0.8
Kaempferol	286.25	41.88	0.64
Ethyl oleate	310.58	32.4	1.43
Squalene	410.8	33.55	2.08
Catechin	290.29	54.83	0.43
β-sitosterol	546.57	33.94	0.44
Butanedioic acid	118.1	39.62	0.44
Epicatechin	290.29	48.96	0.42
Gallic acid	170.13	31.69	0.69
Methyl gallate	184.16	30.91	0.66
4-hydroxybenzaldehyde	122.13	39.98	0.82
5-Dihydroxy-6-methyl-4(H)-pyran-4-one	144.14	37.8	0.48

**Table 3:** The information of 39 potential HILI targets in *Heshouwu*.

Target Gene	Target Protein	Biolocigcal Activity of the Targeted Proteins
RARA	retinoic acid receptor alpha	Adipogenesis
BCL2L1	bcl-2-like protein 1 isoform Bcl-X(L)	hepatic apoptosis
PPARG	peroxisome proliferator-activated receptor gamma	AMPK pathways related fatty acid and glycogen synthesis, and activation of ATP-producing catabolic pathways, such as fatty acid oxidation and glycolysis.
NFE2L2	nuclear factor erythroid 2-related factor 2 isoform 1	Oxidative Stress
RARA	retinoic acid receptor alpha	Adipogenesis
CYP19A1	cytochrome P450,family 19, subfamily A, polypeptide 1	Oxidation stress
MAPT	Microtubule-associated protein tau	AMPK pathways related fatty acid and glycogen synthesis, and activation of ATP-producing catabolic pathways, such as fatty acid oxidation and glycolysis.
MCL1	Myeloid cell leukemia sequence 1	hepatic apoptosis
CYP1A2	cytochrome P450,family 1, subfamily A, polypeptide 2	Oxidation stress
NR1I3	nuclear receptor subfamily 1 group I member 3	lipid metabolism
PPARG	peroxisome proliferator-activated receptor gamma	AMPK pathways related fatty acid and glycogen synthesis, and activation of ATP-producing catabolic pathways, such as fatty acid oxidation and glycolysis.
AHR	aryl hydrocarbon receptor	Adipogenesis
TP53	tumor protein p53	hepatic apoptosis
CASP9	caspase 9	hepatic apoptosis
CASP3	caspase 3	hepatic apoptosis
CASP8	caspase 8	hepatic apoptosis
XBP1	X-box binding protein 1	Transcription factor essential for hepatocyte growth
PPARG	peroxisome proliferator-activated receptor gamma	AMPK pathways related fatty acid and glycogen synthesis, and activation of ATP-producing catabolic pathways, such as fatty acid oxidation and glycolysis.
PPARA	peroxisome proliferator-activated receptor alpha	lipid metabolism
NFE2L2	nuclear factor erythroid 2-related factor 2 isoform 1	Oxidative Stress
MAPT	Microtubule-associated protein tau	AMPK pathways related fatty acid and glycogen synthesis, and activation of ATP-producing catabolic pathways, such as fatty acid oxidation and glycolysis.
PTPN7	tyrosine-protein phosphatase non-receptor type 7 isoform 2	AMPK pathways related fatty acid and glycogen synthesis, and activation of ATP-producing catabolic pathways, such as fatty acid oxidation and glycolysis.
BCL2L1	bcl-2-like protein 1 isoform Bcl-X(L)	hepatic apoptosis
MAPT	Microtubule-associated protein tau	AMPK pathways related fatty acid and glycogen synthesis, and activation of ATP-producing catabolic pathways, such as fatty acid oxidation and glycolysis.
ALOX12	arachidonate 12-lipoxygenase, 12S-type	hepatic inflammation
MMP9	matrix metallopeptidase 9	hepatic fibrosis
CASP3	caspase 3	hepatic apoptosis
MCL1	myeloid cell leukemia sequence 1	hepatic apoptosis
CYP2C8	cytochrome P450,family 2, subfamily C, polypeptide 8	oxidizes a variety of structurally unrelated compounds, including steroids, fatty acids, and xenobiotics
CYP1A1	cytochrome P450, family 1, subfamily A, polypeptide 1	oxidizes a variety of structurally unrelated compounds, including steroids, fatty acids, and xenobiotics
CYP1B1	cytochrome P450, family 1, subfamily B, polypeptide 1	oxidizes a variety of structurally unrelated compounds, including steroids, fatty acids, and xenobiotics
DHCR24	24-dehydrocholesterol reductase	oxidative stress
CCL3	chemokine (C-C motif) ligand 3	hepatic inflammation
SREBF2	sterol regulatory element binding transcription factor 2	lipid metabolism
CASP3	caspase 3	hepatic apoptosis
RXRA	retinoid X nuclear receptor alpha	fatty acid oxidation
PPARG	peroxisome proliferator-activated receptor gamma	AMPK pathways related fatty acid and glycogen synthesis, and activation of ATP-producing catabolic pathways, such as fatty acid oxidation and glycolysis.
PPARD	peroxisome proliferator-activated receptor delta	Adipogenesis

## Supplementary Materials

Supplementary materials are available online. Table S1: Compounds with satisfactory pharmacokinetic properties of XCHT.
