# Supplementary material for: A Network-Based Pharmacology Study of the Herb-Induced Liver Injury Potential of Traditional Hepatoprotective Chinese Herbal Medicines
Source: Molecules. 2017 Apr 14;22(4):632. doi: 10.3390/molecules22040632 (PMC6154655; doi:10.3390/molecules22040632)
Supplement: Supplementary file 1 [file molecules-22-00632-s001.pdf]

Supplemental Table 1. Compounds with satisfactory pharmacokinetic properties of XCHT

Herb	Compound Name	Molecular Weight	Oral Bioavailability (%)	Predicted Caco-2 permeability
Radix Bupleuri	β-Thujene	136.26	46.44	1.82
Radix Bupleuri	19894-97-4	152.26	49.98	1.25
Radix Bupleuri	Alloaromadendrene	204.39	54.04	1.81
Radix Bupleuri	(L)-*α*-Terpineol	154.28	48.8	1.39
Radix Bupleuri	1,8-Cineole	154.28	39.73	1.57
Radix Bupleuri	(-)-Nopinene	136.26	44.84	1.8
Radix Bupleuri	80-57-9	150.24	50.63	1.27
Radix Bupleuri	EIC	280.5	41.9	1.16
Radix Bupleuri	Linoleyl acetate	308.56	42.1	1.36
Radix Bupleuri	Isoliquiritigenin	256.27	85.32	0.44
Radix Bupleuri	(±)-Isoborneol	154.28	86.98	1.27
Radix Bupleuri	D-Camphene	136.26	34.98	1.81
Radix Bupleuri	(*Z*)-caryophyllene	204.39	30.29	1.82
Radix Bupleuri	L-Bornyl acetate	196.32	65.52	1.29
Radix Bupleuri	Pulegone	152.26	51.6	1.39
Radix Bupleuri	(*R*)-Linalool	154.28	39.8	1.33
Radix Bupleuri	Scoparone	206.21	74.75	0.85
Radix Bupleuri	*cis*-Carveol	152.26	45.61	1.39
Radix Bupleuri	Cuparene	202.37	38.26	1.88
Radix Bupleuri	Thymol	150.24	41.47	1.6
Radix Bupleuri	Hexanoic acid	116.18	73.08	0.8
Radix Bupleuri	Methyleugenol	178.25	73.36	1.47
Radix Bupleuri	DEP	222.26	52.19	0.72
Radix Bupleuri	Valerophenone	162.25	42.58	1.46
Radix Bupleuri	BdPh	188.24	42.44	1.32
Radix Bupleuri	(1*R*,4*S*,5*R*)-4-Isopropenyl-1,8-dimethylspiro-[4.5]dec-8-ene	204.39	40.01	1.85
Radix Bupleuri	*p*-Cymen-8-ol	150.24	32.26	1.33
Radix Bupleuri	Amyl benzene	148.27	34.34	1.88
Radix Bupleuri	*cis*-Ligustilide	190.26	51.3	1.3
Radix Bupleuri	Hemosol	136.26	39.84	1.83
Radix Bupleuri	δ-Terpineol	154.28	55.11	1.28
Radix Bupleuri	β-Gurjunene	204.39	51.36	1.81
Radix Bupleuri	L-Limonene	136.26	38.09	1.83
Radix Bupleuri	(*S*)-Carvone	150.24	47.43	1.34
Radix Bupleuri	(-)-Isomenthone	154.28	61.19	1.36
Radix Bupleuri	PTL	86.15	59.53	1.21
Radix Bupleuri	Borneol	154.28	81.8	1.22
Radix Bupleuri	(*Z*,*Z*)-Farnesol	222.41	41.14	1.25

Table S1. *Cont*.

Radix Bupleuri	Eugenol	164.22	56.24	1.35
Radix Bupleuri	*o*-Thymol	150.24	43.28	1.58
Radix Bupleuri	CHEBI:7	136.26	45.2	1.84
Radix Bupleuri	L-Carvone	150.24	49.47	1.35
Radix Bupleuri	Apocynin	166.19	31.71	0.74
Radix Bupleuri	PCR	108.15	51.99	1.56
Radix Bupleuri	WLN: QV4	102.15	70.74	0.78
Radix Bupleuri	Guasol	124.15	51.6	1.28
Radix Bupleuri	IPH	94.12	36.05	1.5
Radix Bupleuri	Undekansaeure	186.33	30.14	0.98
Radix Bupleuri	Amylol	88.17	76.16	1.02
Radix Bupleuri	Nonanoic acid	158.27	40.51	0.92
Radix Bupleuri	Stigmasterol	412.77	43.83	1.44
Radix Bupleuri	Nonanol	144.29	33.19	1.17
Radix Bupleuri	Acoradiene	204.39	36.73	1.85
Radix Bupleuri	Nootkatone	218.37	33.04	1.36
Radix Bupleuri	Methyl naphthalene	142.21	39.01	1.9
Radix Bupleuri	Heptenoic acid	128.19	36.1	0.84
Radix Bupleuri	(2*R*)-2-methylcyclopentan-1-one	98.16	60.04	1.21
Radix Bupleuri	CHEBI:39932	128.24	32.79	1.12
Radix Bupleuri	Nonenoic acid	156.25	65.17	0.95
Radix Bupleuri	2-Octenic acid	142.22	43.49	0.88
Radix Bupleuri	3,5,6,7-Tetramethoxy-2-(3,4,5-trimethoxy-phenyl)chromone	432.46	31.97	0.75
Radix Bupleuri	3,3,5-Trimethylheptane	142.32	47.49	1.8
Radix Bupleuri	ZINC01716732	158.32	33.08	1.26
Radix Bupleuri	(3*R*)-3-Methylcyclotridecan-1-one	210.4	37.65	1.47
Radix Bupleuri	3-Ethyl-2-methyl-1,3-hexadiene	124.25	45.42	1.85
Radix Bupleuri	Heptan-3-one	114.21	68.44	1.29
Radix Bupleuri	(5*S*)-5-Butyloxolan-2-one	142.22	65.08	1.16
Radix Bupleuri	Areapillin	360.34	48.96	0.6
Radix Bupleuri	γ-Undecalactone	184.31	49.12	1.25
Radix Bupleuri	7-Octen-4-ol	128.24	31.46	1.13
Radix Bupleuri	Ayapanin	176.18	41.55	0.97
Radix Bupleuri	8-Nonenoic acid	156.25	52.31	0.94
Radix Bupleuri	Cubebin	356.4	57.13	0.47
Radix Bupleuri	Methyl hexoate	130.21	52.44	1.17
Radix Bupleuri	Octalupine	264.41	47.82	0.48
Radix Bupleuri	PAC	136.16	72.35	0.84
Radix Bupleuri	Sainfuran	286.3	79.91	0.9
Radix Bupleuri	*cis*-2-Undecenal	168.31	47.07	1.39
Radix Bupleuri	(+)-Anomalin	426.5	46.06	0.46

Table S1. *Cont.*

Radix Bupleuri	(5*R*)-5-Isopropyl-2-methyl-1-cyclohex-2-enone	152.26	35.17	1.35
Radix Bupleuri	Cedrenol	220.39	108.56	1.3
Radix Bupleuri	[(3*R*)-3,7-dimethyloct-6-enyl] acetate	198.34	45.54	1.34
Radix Bupleuri	Cyclohexylisocyanate	125.19	41.41	1.1
Radix Bupleuri	Ethyl geranate	196.32	64.07	1.41
Radix Bupleuri	Ethyl protocatechuate	182.19	35.77	0.59
Radix Bupleuri	*cis*-Pinocampheol	154.28	53.92	1.19
Radix Bupleuri	Isopulegol	154.28	50.72	1.24
Radix Bupleuri	Ledol	222.41	82.78	1.32
Radix Bupleuri	Limetin	206.21	36.63	0.88
Radix Bupleuri	Longifolene	204.39	39.49	1.83
Rhizoma Pinelliae	Vanillic acid	168.16	35.47	0.43
Rhizoma Pinelliae	EIC	280.5	41.9	1.16
Rhizoma Pinelliae	Protocatechualdehyde	138.13	38.35	0.43
Rhizoma Pinelliae	Furol	96.09	34.35	1.08
Rhizoma Pinelliae	Zoomaric acid	254.46	35.78	1.18
Rhizoma Pinelliae	24-Ethylcholest-4-en-3-one	412.77	36.08	1.46
Rhizoma Pinelliae	Methyl palmitelaidate	268.49	34.61	1.4
Rhizoma Pinelliae	6-Shogaol	276.41	31	1.07
Rhizoma Pinelliae	Cavidine	353.45	35.64	1.08
Rhizoma Pinelliae	Baicalein	270.25	33.52	0.63
Rhizoma Pinelliae	β-Sitosterol	414.79	36.91	1.32
Rhizoma Pinelliae	Gynesine	137.15	60.07	0.58
Rhizoma Pinelliae	*cis*-Ferulic acid	194.2	54.97	0.53
Rhizoma Pinelliae	*cis*-*p*-Coumarate	164.17	45.98	0.46
Rhizoma Pinelliae	Linolenic acid	278.48	45.01	1.21
Rhizoma Pinelliae	Stigmasterol	412.77	43.83	1.44
Rhizoma Pinelliae	Anethole	148.22	32.49	1.75
Rhizoma Pinelliae	Gondoic acid	310.58	30.7	1.2
Rhizoma Pinelliae	Coniferin	314.41	31.11	0.42
Rhizoma Pinelliae	Eciphin	165.26	43.35	0.92
Rhizoma Pinelliae	Oleic acid	282.52	33.13	1.17
Rhizoma Pinelliae	l-Pseudoephedrine	165.26	45.01	1.06
Rhizoma Pinelliae	Octylene	112.24	39.25	1.79
Rhizoma Pinelliae	10,13-Eicosadienoic acid	308.56	39.99	1.22
Rhizoma Pinelliae	8-Octadecenoic acid	282.52	33.13	1.15
Rhizoma Pinelliae	BVE	100.18	42.32	1.44
Rhizoma Pinelliae	(3*S*,6*S*)-3-(Benzyl)-6-(4-hydroxybenzyl)-piperazine-2,5-quinone	310.38	46.89	0.41
Rhizoma Pinelliae	Cycloartenol	426.8	38.69	1.53
Rhizoma Pinelliae	2*Z*-Hexadecenoic acid	254.46	34.02	1.22

Table S1. *Cont*.

Rhizoma Pinelliae	Valeraldoxime	101.17	82.58	0.72
Scutellariae Radix	1,8-Cineole	154.28	39.73	1.57
Scutellariae Radix	PEL	122.18	44.03	1.11
Scutellariae Radix	Acacetin	284.28	34.97	0.67
Scutellariae Radix	Wogonin	284.28	30.68	0.79
Scutellariae Radix	(±)-Isoborneol	154.28	86.98	1.27
Scutellariae Radix	(*R*)-linalool	154.28	39.8	1.33
Scutellariae Radix	BOX	121.12	31.55	0.54
Scutellariae Radix	(2*R*)-7-Hydroxy-5-methoxy-2-phenyl-chroman-4-one	270.3	55.23	0.87
Scutellariae Radix	Eugenol	164.22	56.24	1.35
Scutellariae Radix	β-Patchoulene	204.39	50.69	1.79
Scutellariae Radix	Baicalein	270.25	33.52	0.63
Scutellariae Radix	5,8,2'-Trihydroxy-7-methoxyflavone	300.28	37.01	0.76
Scutellariae Radix	Dihydrobaicalin_qt	272.27	40.04	0.56
Scutellariae Radix	Salvigenin	328.34	49.07	0.86
Scutellariae Radix	5,2',6'-Trihydroxy-7,8-dimethoxyflavone	330.31	45.05	0.48
Scutellariae Radix	5-(2-Hydroxyethyl)-2-methoxyphenol	168.21	31.95	0.6
Scutellariae Radix	Dihydrooroxylin A	286.3	38.72	0.71
Scutellariae Radix	Skullcapflavone II	374.37	69.51	0.68
Scutellariae Radix	Oroxylin A	284.28	41.37	0.76
Scutellariae Radix	Tyrosol	138.18	33.81	0.65
Scutellariae Radix	Panicolin	314.31	76.26	0.84
Scutellariae Radix	5,7,4'-Trihydroxy-8-methoxyflavone	300.28	36.56	0.46
Scutellariae Radix	Neobaicalein	374.37	104.34	0.74
Scutellariae Radix	Dihydrooroxylin	286.3	66.06	0.67
Scutellariae Radix	β-Sitosterol	414.79	36.91	1.32
Scutellariae Radix	Sitosterol	414.79	36.91	1.32
Scutellariae Radix	Norwogonin	270.25	39.4	0.6
Scutellariae Radix	5,2'-Dihydroxy-6,7,8-trimethoxyflavone	344.34	31.71	0.93
Scutellariae Radix	(-)-*α*-Cedrene	204.39	55.56	1.81
Scutellariae Radix	(*S*)-Matsutake alcohol	128.24	40.11	1.19
Scutellariae Radix	L-Menthone	154.28	57.9	1.35
Scutellariae Radix	EIC	280.5	41.9	1.16
Scutellariae Radix	Stigmasterol	412.77	43.83	1.44
Scutellariae Radix	DBP	278.38	64.54	0.8
Scutellariae Radix	WLN: VHR	106.13	32.63	1.32
Scutellariae Radix	Hyacinthin	120.16	38.65	1.31
Scutellariae Radix	D-Isomenthone	154.28	61.2	1.35
Scutellariae Radix	*p*-Coumaric acid	164.17	43.29	0.46
Scutellariae Radix	Coptisine	320.34	30.67	1.21

Table S1. *Cont*.

Scutellariae Radix	bis[(2*S*)-2-Ethylhexyl]benzene-1,2-dicarboxylate	390.62	43.59	0.98
Scutellariae Radix	Supraene	410.8	33.55	2.08
Scutellariae Radix	Hypnon	120.16	48.19	1.36
Scutellariae Radix	Methyl palmitelaidate	268.49	34.61	1.4
Scutellariae Radix	Methyl linolelaidate	294.53	41.93	1.46
Scutellariae Radix	Pulegone	152.26	51.6	1.39
Zingiber Officinale	(L)-α-Terpineol	154.28	48.8	1.39
Zingiber Officinale	ZINC02040970	222.41	40.43	1.44
Zingiber Officinale	Bornyl acetate	196.32	65.55	1.3
Zingiber Officinale	1,8-Cineole	154.28	39.73	1.57
Zingiber Officinale	(*S*)-*p*-Mentha-1,8-dien-7-al	150.24	39	1.36
Zingiber Officinale	Calarene	204.39	52.16	1.82
Zingiber Officinale	Moslene	136.26	33.02	1.88
Zingiber Officinale	Thymol	150.24	41.47	1.6
Zingiber Officinale	Isoeugenol	164.22	70.1	1.38
Zingiber Officinale	L-Limonene	136.26	38.09	1.83
Zingiber Officinale	6-Gingerol	294.43	35.64	0.54
Zingiber Officinale	6-Shogaol	276.41	31	1.07
Zingiber Officinale	β-Rhodinol	156.3	38.05	1.19
Zingiber Officinale	β-Citronellol	156.3	38.89	1.2
Zingiber Officinale	(1*S*,5*S*)-1-isopropyl-4-methylenebicyclo-[3.1.0]hexane	136.26	46.21	1.83
Zingiber Officinale	β-Sitosterol	414.79	36.91	1.32
Zingiber Officinale	[(3*S*)-3,7-dimethylocta-1,6-dien-3-yl] acetate	196.32	36.84	1.4
Zingiber Officinale	Terpinen-4-ol	154.28	81.41	1.36
Zingiber Officinale	HEPTENE	98.21	50.67	1.79
Zingiber Officinale	Zingiberol	222.41	37.24	1.36
Zingiber Officinale	6-Methylgingediacetate	394.56	48.73	0.55
Zingiber Officinale	Vanillin	152.16	52	0.68
Zingiber Officinale	Alloaromadedrene	204.39	53.46	1.83
Zingiber Officinale	(-)-Citronellal	154.28	35.71	1.34
Zingiber Officinale	D-Linalool	154.28	38.29	1.29
Zingiber Officinale	(±)-Isoborneol	154.28	86.98	1.27
Zingiber Officinale	Hemosol	136.26	39.84	1.83
Zingiber Officinale	α-Longipinene	204.39	53.26	1.83
Zingiber Officinale	Vanillic acid	168.16	35.47	0.43
Zingiber Officinale	(~)-α-Pinene	136.26	46.25	1.85
Zingiber Officinale	CAM	152.26	67.17	1.29
Zingiber Officinale	EIC	280.5	41.9	1.16

Table S1. *Cont*.

Zingiber Officinale	Guaiol	222.41	38.77	1.36
Zingiber Officinale	L-Bornyl acetate	196.32	65.52	1.29
Zingiber Officinale	(*R*)-linalool	154.28	39.8	1.33
Zingiber Officinale	Aromadendrene	204.39	55.74	1.81
Zingiber Officinale	Borneol	154.28	81.8	1.22
Zingiber Officinale	3,4-Dimethylstyrene	132.22	32.41	1.9
Zingiber Officinale	Eugenol	164.22	56.24	1.35
Zingiber Officinale	(~)-β-Phellandrene	136.26	40.44	1.83
Zingiber Officinale	*o*-Thymol	150.24	43.28	1.58
Zingiber Officinale	β-Cubebene	204.39	32.81	1.83
Zingiber Officinale	L-Carvone	150.24	49.47	1.35
Zingiber Officinale	Linolenic acid	278.48	45.01	1.21
Zingiber Officinale	Stigmasterol	412.77	43.83	1.44
Zingiber Officinale	(~)-Epoxycaryophyllene	220.39	35.94	1.57
Zingiber Officinale	TMH	136.26	46.25	1.82
Zingiber Officinale	Neryl acetate	196.32	57.47	1.25
Zingiber Officinale	(~)-*α*-Cedrene	204.39	55.56	1.81
Zingiber Officinale	Hexanal	100.18	55.71	1.25
Zingiber Officinale	DBP	278.38	64.54	0.8
Zingiber Officinale	*m*-Cymol	134.24	48.85	1.88
Zingiber Officinale	Nerol	154.28	35.66	1.15
Zingiber Officinale	2-Heptanone	114.21	46.56	1.31
Zingiber Officinale	*o*-Cymol	134.24	51.89	1.88
Zingiber Officinale	L-Menthone	154.28	57.9	1.35
Zingiber Officinale	OXA	46.08	63.21	0.76
Zingiber Officinale	Furanodiene	216.35	45.11	1.77
Zingiber Officinale	β-Pinene	136.26	44.77	1.85
Zingiber Officinale	Germacrone	218.37	32.5	1.33
Zingiber Officinale	Terpilene	136.26	33.95	1.84
Zingiber Officinale	(5*S*)-1-Isopropyl-4-methylbicyclo[3.1.0]-hex-3-ene	136.26	47.13	1.81
Zingiber Officinale	Car-3-ene	136.26	45.15	1.85
Zingiber Officinale	(*R*)-*p*-Menth-1-en-4-ol	154.28	32.16	1.33
Zingiber Officinale	Hepanal	204.39	53.83	1.86
Zingiber Officinale	Calarene	204.39	51.55	1.82
Zingiber Officinale	(1*R*,4*S*,4a*R*,8a*R*)-4-Isopropyl-1,6-dimethyl-3,4,4a,7,8,8a-hexahydro-2*H*-naphthalen-1-ol	222.41	31.67	1.32
Zingiber Officinale	ZINC00388662	196.32	67.05	1.24
Zingiber Officinale	β-Thujene	136.26	46.44	1.82
Zingiber Officinale	L-Verbenone	150.24	50.66	1.26

Table S1. *Cont*.

Zingiber Officinale	(1*S*,2*S*)-2-Isopropenyl-4-isopropylidene-1-methyl-1-vinylcyclohexane	204.39	34.47	1.87
Zingiber Officinale	Alloaromadendrene	204.39	54.04	1.81
Zingiber Officinale	*cis*-Lanceol	220.39	35.94	1.33
Zingiber Officinale	(1*S*,5*R*)-7,7-dimethyl-4-bicyclo[3.1.1]hept-3-ene carboxaldehyde	150.24	41.25	1.34
Jujubae Fructus	Vaccenic acid	282.52	33.13	1.17
Jujubae Fructus	Stepharine	297.38	31.55	0.64
Jujubae Fructus	Sylvestrene	136.26	51.28	1.82
Jujubae Fructus	Xizyphus saponin I_qt	472.78	32.69	0.42
Jujubae Fructus	Daechualkaloid A	139.17	34.94	0.62
Jujubae Fructus	Jujuboside A_qt	472.78	36.67	0.54
Jujubae Fructus	Coumestrol	268.23	32.49	0.55
Jujubae Fructus	Daechuine S7	514.74	44.82	0.46
Jujubae Fructus	Mauritine D	342.46	89.13	0.59
Jujubae Fructus	WLN: Q1R	108.15	58.68	1.08
Jujubae Fructus	Berberine	336.39	36.86	1.24
Jujubae Fructus	(*S*)-Coclaurine	285.37	42.35	0.7
Jujubae Fructus	Zoomaric acid	254.46	35.78	1.18
Jujubae Fructus	IES	175.2	46.15	0.7
Jujubae Fructus	Mairin	456.78	55.38	0.73
Jujubae Fructus	Stigmasterol	412.77	43.83	1.44
Jujubae Fructus	Ziziphin_qt	472.78	66.95	0.49
Jujubae Fructus	β-Sitosterol	414.79	36.91	1.32
Jujubae Fructus	Stepholidine	327.41	33.11	0.83
Jujubae Fructus	Oleic acid	282.52	33.13	1.17
Jujubae Fructus	Nuciferin	295.41	34.43	1.22
Jujubae Fructus	Linoleic	280.5	41.9	1.23
Jujubae Fructus	*p*-Coumaric acid	164.17	43.29	0.46
Jujubae Fructus	Protoporphyrin	562.72	30.86	0.67
Jujubae Fructus	Fumarine	353.4	59.26	0.56
Jujubae Fructus	Moupinamide	313.38	86.71	0.55
Jujubae Fructus	β-Carotene	536.96	37.18	2.25
Licorice	*o*-Xylene	106.18	45.55	1.85
Licorice	*m*-Xylene	106.18	47.43	1.83
Licorice	*p*-Xylene	106.18	48.74	1.83
Licorice	(L)-α-Terpineol	154.28	48.8	1.39
Licorice	Inermine	284.28	75.18	0.89
Licorice	α-Cubebol	208.38	64.81	1.32
Licorice	ICO	161.17	33.86	0.82
Licorice	Isoliquiritigenin	256.27	85.32	0.44
Licorice	DFV	256.27	32.76	0.51

Table S1. *Cont.*

Licorice	Izoforon	138.23	44.98	1.28
Licorice	Mairin	456.78	55.38	0.73
Licorice	Isoheptane	100.23	59.94	1.81
Licorice	Heptane	100.23	41.8	1.77
Licorice	Glycyrol	366.39	90.78	0.71
Licorice	Jaranol	314.31	50.83	0.61
Licorice	21987_FLUKA	136.26	40.92	1.84
Licorice	Medicarpin	270.3	49.22	1
Licorice	EB	106.18	49.38	1.83
Licorice	Pinocembrin	256.27	64.72	0.61
Licorice	Sitosterol	414.79	36.91	1.32
Licorice	Lupiwighteone	338.38	51.64	0.68
Licorice	7-Methoxy-2-methylisoflavone	266.31	42.56	1.16
Licorice	Formononetin	268.28	69.67	0.78
Licorice	2-Caren-10-al	150.24	44.74	1.37
Licorice	Calycosin	284.28	47.75	0.52
Licorice	β-Terpinene	136.26	42.29	1.85
Licorice	Anethole	148.22	32.49	1.75
Licorice	(*E*)-1-Butoxyhex-2-ene	156.3	41.72	1.5
Licorice	(2*S*)-2-[4-Hydroxy-3-(3-methylbut-2-enyl) phenyl]-8,8-dimethyl-2,3-dihydropyrano-[2,3-f]chromen-4-one	390.51	31.79	1
Licorice	Euchrenone	406.56	30.29	1.09
Licorice	Glyasperin B	370.43	65.22	0.47
Licorice	Glyasperin F	354.38	75.84	0.43
Licorice	Glyasperin C	356.45	45.56	0.71
Licorice	Isotrifoliol	298.26	31.94	0.53
Licorice	(*E*)-1-(2,4-Dihydroxyphenyl)-3-(2,2-dimethylchromen-6-yl)prop-2-en-1-one	322.38	39.62	0.66
Licorice	Kanzonols W	336.36	50.48	0.63
Licorice	Semilicoisoflavone B	352.36	48.78	0.45
Licorice	Glepidotin A	338.38	44.72	0.79
Licorice	Glepidotin B	340.4	64.46	0.46
Licorice	Octadiene	110.22	34.53	1.81
Licorice	WLN: 4OVR	178.25	48.41	1.31
Licorice	Phaseolinisoflavan	324.4	32.01	1.01
Licorice	Glypallichalcone	284.33	61.6	0.76
Licorice	Karenzu DK2	224.27	62.26	0.94
Licorice	8-(6-Hydroxy-2-benzofuranyl)-2,2-dimethyl-5-chromenol	308.35	58.44	1
Licorice	(1*S*,2*S*)-1,2-Dimethylcyclopentane	98.21	41.78	1.78
Licorice	Licochalcone B	286.3	76.76	0.47

Licorice	2,2-Dimethylpentane	100.23	55.33	1.79
Licorice	Licochalcone G	354.43	49.25	0.64
Licorice	3-(2,4-Dihydroxyphenyl)-8-(1,1-dimethyl-prop-2-enyl)-7-hydroxy-5-methoxy-coumarin	368.41	59.62	0.4
Licorice	Licoricone	382.44	63.58	0.53
Licorice	Gancaonin A	352.41	51.08	0.8
Licorice	Gancaonin B	368.41	48.79	0.58
Licorice	2,3-Dimethylhexane	114.26	46.24	1.78
Licorice	3-(3,4-Dihydroxyphenyl)-5,7-dihydroxy-8-(3-methylbut-2-enyl)chromone	354.38	66.37	0.52
